# Erythema multiforme-like lip presentation in pemphigus vulgaris patients: a multicenter case series

**DOI:** 10.1186/s12903-023-03665-w

**Published:** 2023-12-01

**Authors:** Ghidaa Subahi, Sara Waheeb, Nada Binmadi, Soulafa Almazrooa, Sara Akeel, Jenny Öhman, Amal Dafar

**Affiliations:** 1 Oral Medicine and Pathology Saudi Board Program, Jeddah, Saudi Arabia; 2 Magrabi Hospitals, Jeddah, Saudi Arabia; 3 Oral Diagnostic Sciences Department, Faculty of Dentistry, King Abdulaziz University, Jeddah, Saudi Arabia; 4 Department of Oral Medicine and Pathology, Institute of Odontology, Sahlgrenska Academy, University of Gothenburg, Gothenburg, Sweden; 5 Department of Pathology, Sahlgrenska University Hospital, Gothenburg, Sweden; 6 Department of Oral and Maxillofacial Surgery, King Fahad General Hospital, Jeddah, Saudi Arabia

**Keywords:** Pemphigus vulgaris, Erythema multiforme, Oral lesions, Lip crusting, Lip hemorrhage

## Abstract

Pemphigus vulgaris (PV) is a chronic autoimmune mucocutaneous blistering disease. Autoantibodies are directed against desmogleins, leading to the formation of intraepithelial bullae. PV, as with other autoimmune mucocutaneous disorders of the oral cavity, presents diagnostic and therapeutic challenges. Approximately 50–70% of cases present first with oral lesions. The lesions commonly start as vesicles or bullae that rapidly rupture, leading to erosions and ulcerations. The palatal, gingival, buccal, and labial mucosa are the most commonly affected sites. Oral PV can mimic several other diseases that cause mucosal erosions and/or ulcerations, including erythema multiforme (EM). EM is an acute, immune-mediated, self-limited hypersensitivity condition primarily associated with herpes simplex infection. Oral lesions can be variable, but a very characteristic presentation with labial hemorrhagic erosions, ulcerations and crusting is commonly seen. In this case series, we present six cases of PV: one male patient and five female patients whose ages ranged from 34 to 65 years old. All patients presented with hemorrhage and crusting of the lips in addition to multiple intraoral erosions and ulcerations. Three patients presented with oral and skin lesions. All patients underwent biopsies, and a diagnosis of PV was confirmed. All patients were treated with steroids (topical and systemic) and variable steroid-sparing agents. This case series emphasizes that oral PV may be misdiagnosed as EM in a subgroup of patients who present with persistent lip hemorrhage and crusting. Therefore, a comprehensive history, clinical examination and incisional biopsies should be considered in such patients.

## Introduction

The term pemphigus encompasses a group of chronic potentially life-threatening autoimmune mucocutaneous diseases characterized by blistering of cutaneous surfaces, mucosal surfaces or both [1]. Several subtypes of pemphigus have been identified, including pemphigus vulgaris, pemphigus vegetans, pemphigus foliaceus, pemphigus erythematosus, paraneoplastic pemphigus and IgA pemphigus [2]. Pemphigus vulgaris (PV) is the most common variant that affects the oral cavity. In PV, serum autoantibodies (IgG) are directed against desmosomal components (desmoglein 1 and/or desmoglein 3) that adhere keratinocytes to each other [3]. Subsequently, keratinocyte separation, acantholysis, and intraepithelial splitting occur, which manifest clinically as cutaneous and/or mucosal blisters [1]. Approximately 70.0% of cases exhibit oral signs that precede skin or other mucosal lesions [4]. Exclusive oral involvement may be seen in almost 50.0% of PV cases [5]. Oral lesions of PV start as multiple vesicles and/or bullae, which are rarely observed due to the fragile nature of the bullae and the mechanical stress caused during the daily activities of eating and speaking [6]. Rupture of vesicles or bullae leads to painful widespread erosions and ulcerations that primarily affect the buccal mucosa, tongue, palate and lips, which eventually heal without scarring [6]. Desquamative gingivitis can be seen in severe cases of PV [7–9]. For PV diagnosis, clinicians must correlate disease history with the clinical and histopathological findings. Direct immunofluorescence (DIF) is used for the definitive diagnosis of PV, which demonstrates characteristic intercellular deposits of IgG and C3 in the epithelium layer [1]. Indirect immunofluorescence (IIF) may be useful for monitoring disease activity [10].

Oral PV may mimic several diseases that cause mucosal erosions and/or ulcerations. Erythema multiforme (EM) is one of those mimickers. EM is an acute, mucocutaneous, immune-mediated self-limiting condition that often resolves within a few weeks without mention of sequalae. EM commonly affects young adults between the second and fifth decades with no specific gender predication [11, 12]. The pathogenesis of EM has been suggested to be a type IV hypersensitivity reaction mediated by cytotoxic T cells [13]. Numerous factors may trigger such a reaction, including infection with Human adenovirus (HAdV), HSV-1 and HSV-2 [14–16]. Mycoplasma pneumoniae was found to be associated with EM, especially in children [17, 18]. Drug-induced EM is reported in approximately 10.0% of cases and is most commonly associated with the use of nonsteroid anti-inflammatory drugs, sulfonamides, antiepileptics, and antibiotics [19]. The spectrum and classification of EM have been discussed based on the clinical course [20].

Oral mucosal involvement in EM may precede other lesions or may arise in isolation [13]. The typical oral presentation of EM includes diffuse shallow ulcerations and/or erosions commonly in the anterior part of the oral cavity and primarily affects the nonkeratinized mucosa. Lip involvement typically presents as swelling, cracking and hemorrhagic crusting [12]. The diagnosis of EM is based entirely on the clinical presentation and patient’s history, with no specific test to confirm the diagnosis. However, a biopsy may be indicated in atypical cases of EM to exclude other differential diagnoses, such as pemphigus, pemphigoid and others [21]. Microscopic examination of EM lesions is characterized by inflammatory infiltration of the basement membrane with the presence of T cells and macrophages. Intra- and subepithelial bullae formation may result from necrosis in the basal and supra-basal layers, and DIF examination may illustrate nonspecific deposition of C3 and fibrin along the basement membrane [13]. EM oral mucosal lesions can be treated with topical high-potency steroids for cases with minimal mucosal involvement. However, patients with more severe lesions may require systemic steroid therapy [21].

Oral PV may be misdiagnosed as EM in a subgroup of patients presenting with lip hemorrhage and crusting. Therefore, this case series emphasizes that oral PV may be misdiagnosed as EM. This series highlights the importance of a comprehensive history, clinical examination and incisional biopsies for this subgroup of patients.

## Case report

We retrospectively reviewed six cases of PV presenting with hemorrhage lip crusting that had been treated at three different institutions in Saudi Arabia and Sweden between 2015 and 2022. Table 1 summarizes the demographics, lip presentation, intraoral lesion sites, histopathological and DIF findings, management and follow-up duration. Informed consent was obtained from all patients, and this study was approved by the Institutional Review Board (IRB), Ministry of Health, Jeddah, Saudi Arabia (approval no. A01372).

**Table 1:** Demographics, lip presentation, intraoral lesion sites, histopathological and DIF findings, management and follow-up duration

Case #	Age (years)	Gender	Lips presentation	Intraoral sites of erosions and ulcers	H&E (cases 1–6)	DIF (intercellular)	Management	Follow-up (months)
**Case 1**	34	Female	Erosions and ulcers on the lower lip (Fig. 1a)	Buccal mucosa bilaterally, hard palate, soft palate, and attached gingivae	Supra-basal clefting, acantholysis, and tombstone appearance of basal cell layer (Fig. 2a and f)	Positive for IgG	P, AZA, N	14
**Case 2**	65	Female	Erosions and crusting on both upper and lower lips (Fig. 1b)	Buccal mucosa bilaterally, lateral border of the tongue and floor of the mouth		Positive for IgG	P, AZA, DEXA, N	32
**Case 3**	33	Female	Erosions and crusting on both upper and lower lips (Fig. 1c)	Lateral borders of the tongue, ventral surface of the tongue, soft palate, and throat		Positive for IgG and C3	P, C, N	84
**Case 4**	64	Female	Erosions and crusting on the lower lip (Fig. 1d)	Lateral borders of the tongue, palate and lower anterior attached gingivae labially		NA	P, AZA, P-MW	60
**Case 5**	37	Male	Erosions, ulcers and crusting on both upper and lower lips (Fig. 1e)	Buccal mucosa bilaterally, ventral surface of the tongue, floor of the mouth		NA	P, AZA, P-MW	5
**Case 6**	57	Female	Erosions, ulcers and crusting on both upper and lower lips (Fig. 1f)	Widespread intraoral ulcerations and erosions		NA	P, P-MW	NA

DIF, Direct immunofluorescence; P, prednisolone; P-MW; prednisolone mouthwash, AZA, azathioprine; DEXA; topical dexamethasone (0.5 mg/5 ml) mouthwash; N, nystatin mouthwash; C, clobetasol propionate oral gel 0.025%; NA, not applicable

### Case 1

**Fig. 1:**
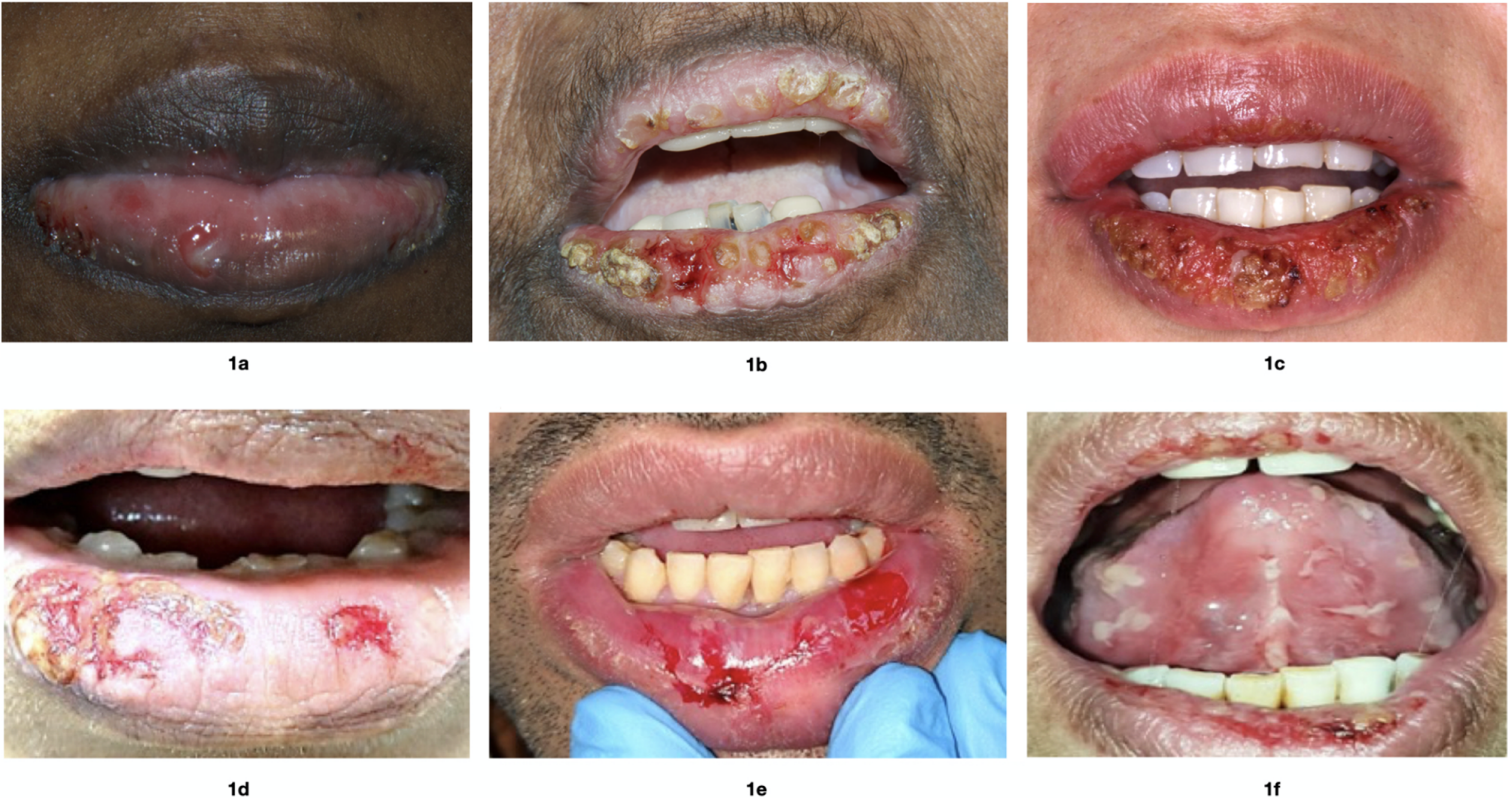
Clinical presentation of lip lesions in cases 1–6 (**a-f**)

**Fig. 2:**
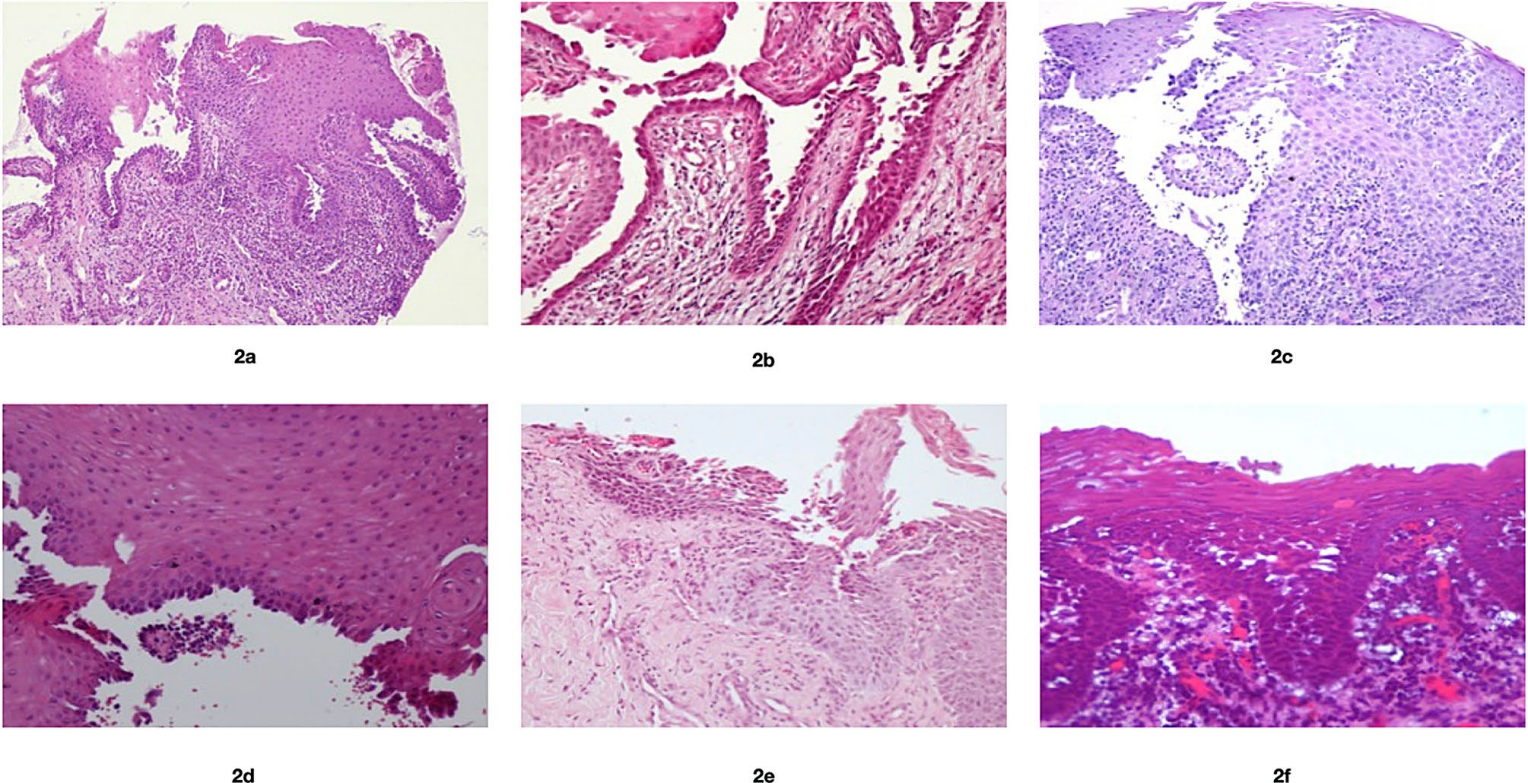
Classic (H&E stain) histopathological findings under 20x magnification for pemphigus vulgaris demonstrate suprabasal clefting, acantholysis and tombstone appearance of the basal cell layer in cases 1–6 (**a-f**). H&E; hematoxylin and eosin

A 34-year-old Saudi female patient was referred to the Oral Medicine Clinic at King Fahad General Hospital, Jeddah, Saudi Arabia, by a dermatologist. She complained of pain and a burning sensation in her mouth that started 6 months prior. Regarding her medical history, she had iron-deficiency anemia for 20 years. Accordingly, she took ferrous sulfate (100 mg) and folic acid (1 mg), both once daily. Clinical examination revealed erosions and ulcers in the lower lip, buccal mucosa bilaterally, hard and soft palate, and attached gingivae (Fig. 1a). The patient had skin lesions on the abdomen and right nostril. Our tentative differential diagnoses were PV, mucous membrane pemphigoid, and EM. A 4-mm punch biopsy was taken from the perilesional area of the right buccal mucosa. Histopathological examination using hematoxylin and eosin (H & E) revealed supra-basal acantholysis and a tombstone appearance of the basal cell layer, which was suggestive of PV (Fig. 2a). Subsequently, another incisional biopsy was taken for direct immunofluorescence (DIF) examination, which demonstrated intercellular deposition of IgG. Thus, the diagnosis of PV was confirmed. The patient was started on treatment with prednisolone 1 mg/kg/day (50 mg). Approximately one month after the initiation of steroid therapy, the patient achieved remission of her oral lesions. Thus, gradual steroid tapering (10 mg every 10 days) was started, and azathioprine (AZA) 50 mg once daily was added. The patient continued her treatment with tapering of steroids and AZA. Because the patient was susceptible to developing oral candidiasis during treatment with systemic steroids, nystatin oral suspension 100,000 IU/ml was used as a mouthwash to first treat and then as a prophylactic against oral candidiasis. The total treatment period was nine months. Thereafter, all medications were discontinued, and the patient was free from subjective symptoms and objective signs for fourteen months.

### Case 2

A 65-year-old Saudi female patient was referred to the Oral Medicine Clinic at King Fahad General Hospital, Jeddah, Saudi Arabia, by a rheumatologist. She complained of ulcers on her tongue and lips. With respect to her medical history, she had developed severe oral ulcerations six years prior, which waxed and waned. Recently, she had developed genital lesions; hence, the rheumatologist established the diagnosis of Behcet’s disease. Lately, she developed skin lesions that healed before her first visit to our clinic. On her first visit, she was taking prednisolone 5 mg once daily, azathioprine (AZA) 150 mg once daily, and colchicine 0.5 mg once daily. Adalimumab was administered for 7 months without substantial benefit and was discontinued 8 months before the visit. Clinical examination revealed erosions and crusting on both the upper and lower lips (Fig. 1b). Intraorally, ulceration was seen on the buccal mucosa bilaterally, floor of the mouth, and left lateral border of the tongue. Our tentative diagnoses were PV and EM. Two incisional biopsies were obtained and examined by routine H&E and DIF, which demonstrated similar findings as in Case 1 (Fig. 2b). Therefore, the diagnosis of PV was confirmed. The patient was started on prednisolone 1 mg/kg/d (50 mg) and advised to continue azathioprine 150 mg once daily. Approximately 6 weeks after the initiation of prednisolone treatment, the oral lesion went into the remission phase, and prednisolone was tapered gradually (10 mg every 10 days). During the follow-up period, the patient had several exacerbations in which the systemic steroid dose was adjusted based on the clinical findings and the response to therapy. Furthermore, topical steroid mouthwash (dexamethasone 0.5 mg/5 ml) and nystatin oral suspension as mouthwash 100.000 IU/ml were used frequently.

### Case 3

A 33-year-old female patient of Syrian descent presented to the Clinic of Oral Medicine, Public Dental Health, Gothenburg, Sweden, Region Västra Götaland of Sweden. She complained of painful ulcers in the oral cavity and lips that started seven months prior, accompanied by a feeling of burning sensation, itching, and pain. The patient also complained of dysphagia, which prompted her to seek out an ENT physician. Upon examination by ENT, ulcers were observed at the epiglottis. The patient had no significant medical history but was admitted for investigation of suspected Behçet’s disease. Clinical examination revealed ulcerations on the ventral surface of the tongue and lateral borders of the tongue, palate, and throat. The lip lesions presented as erosions coated with crusting (Fig. 1c). Our tentative diagnosis was EM with secondary infected ulcers and PV. A sample for microbiology examination was taken from the outer surface of the lower lip. This sample demonstrated the presence of *Staphylococcus aureus*. For the treatment of the lip lesions, topical retapamulin 10 mg/g for 5 days and topical hydrogen peroxide 1% for one week, both twice daily, were prescribed. A 4-mm punch biopsy was taken from the perilesional area of the upper labial mucosa. Routine H & E examinations revealed findings similar to those in Case 1, suggesting PV (Fig. 2c). Subsequently, another 4-mm punch biopsy was taken from the lower labial mucosa for DIF examination, which demonstrated intercellular deposition of IgG and C3 in a net-like pattern. Indirect IF revealed the presence of antibodies against desmoglein 3. Blood samples were negative for antibodies against envoplakin, thus ruling out paraneoplastic pemphigus. Accordingly, the diagnosis of PV was confirmed, and the patient was then referred to the dermatology department for management. Her initial treatment regimen included prednisolone 0.5 mg/kg (30 mg per day), omeprazole 20 mg and Calcichew-D forte 500 mg as osteoporosis prophylaxis per day. The patient complained of discomfort in the eyes and genital mucosa, but no manifestation of PV was seen in these locations. Approximately two months after the initiation of steroid therapy, the patient achieved remission of her oral lesions. After remission, the prednisolone dose was decreased by 5 mg per week but with some plateau effect. One year after the initiation of prednisolone treatment, the dose was 5 mg per day. The patient continued her treatment with tapering steroids, and after seven years, the patient was taking 2.5 mg prednisolone every other day in combination with Calcichew-D 500 mg daily. The Dexa scan showed no signs of osteoporosis. Topical treatment with clobetasol propionate 0.025% oral gel in combination with local antifungal medication Nystimex oral suspension 10.0000 IU/ml was prescribed for use when exacerbation occurred. The patient did not have any skin lesions during the seven-year follow-up period.

### Case 4

A 64-year-old Yemeni female presented to the Oral Medicine Clinic of the Faculty of Dentistry at King Abdulaziz University, Jeddah, Saudi Arabia. Her chief complaint was ulceration of the lip, tongue and buccal mucosa. Her medical history was significant for hypertension, hyperlipidemia, and hypothyroidism. She was taking irbesartan 150 mg/12.5 mg, atenolol 50 mg, rosuvastatin 10 mg, levothyroxine 150 mg, Calcos Vit. D3 500 mg/400 IU, pregabalin, Jus aspirin 81 mg, acyclovir 200 mg/5 ml and topical hyaluronic acid. Clinical examination revealed erosions and crusting of the lower lip. Intraorally, ulceration was seen on the palatal mucosa, lateral borders of the tongue and lower anterior attached gingiva labially (Fig. 1d). The provisional diagnosis was PV. An incisional biopsy was taken from the palate for routine H&E examination, which revealed findings comparable to those of Case 1 (Fig. 2d). Thus, the diagnosis of PV was confirmed. For treatment, prednisolone syrup 15 mg/5 ml as a mouthwash three times daily and prednisolone 1 mg/kg/d (60 mg) were initiated, followed by azathioprine 100 mg once daily. Follow-up was performed every 2 weeks initially for 2 months and then every 3 months. The patient’s condition was controlled after 3 months, and systemic prednisolone was tapered over 6 months. The patient remained on azathioprine for 18 months, after which she developed fatigue and requested discontinuation of the medication. The patient was subsequently controlled with a topical steroid rinse. The last follow-up was performed 5 years after the initial diagnosis.

### Case 5

A 37-year-old Saudi male patient presented to the Oral Medicine Clinic at the Faculty of Dentistry at King Abdulaziz University, Jeddah, Saudi Arabia. He complained of pain while eating and drinking, in addition to a limited ability to open his mouth for the last two months. The patient had no significant medical history. He was a smoker who had used approximately 10 cigarettes per day for the last 12 years. Upon clinical examination, ulcerations were observed on the upper and lower lips with crusting. Erosions were observed bilaterally on the ventral surface of the tongue, floor of the mouth and buccal mucosa (Fig. 1e). An incisional biopsy was taken from the lower lip for routine H&E examination, which was also suggestive of PV, as in the previous cases (Fig. 2e). Hence, a diagnosis of PV was established. Prednisolone 45 mg/day was initiated and continued for two weeks, and the dose was subsequently tapered over 3 months. In addition to systemic treatment, topical treatment was added using prednisolone syrup 15 mg/5 ml as a mouthwash thrice daily for 3 months. Two months later, he presented with a relapse of intraoral lesions in which azathioprine 50 mg, once daily, was added to the preexisting steroid mouthwash. Follow-up was performed every 2 weeks for 2 months and then every 3 months. The patient was then lost to follow-up.

### Case 6

A 57-year-old Saudi female patient presented to the Oral Medicine Clinic at the Faculty of Dentistry at King Abdulaziz University, Jeddah, Saudi Arabia. The patient’s main complaints were pain and labial ulcerations. She had hypothyroidism for which she was taking levothyroxine. Clinical examination revealed widespread ulcerations and erosions intraorally. The upper and lower lips exhibited erosions, ulcerations and crusting (Fig. 1f). Extraorally, skin lesions were observed around the armpit. The patient also had genital lesions. An incisional biopsy was taken from the lower labial mucosa for routine H&E examination, which demonstrated findings consistent with PV (Fig. 2f). The patient started treatment with prednisolone 40 mg once daily as well as topical treatment with prednisolone syrup 15 mg/5 ml as a mouthwash thrice daily for 3 months. During multiple follow-up visits, the patient relapsed once steroids were tapered, and she was referred to a dermatologist at a tertiary care center for rituximab therapy. She was treated and followed up there.

## Discussion

In the current case series, we reported six patients with oral PV who presented first with a clinical picture that mimicked EM. The anterior part of the oral cavity was affected by lip swelling, hemorrhage and crusting. The intraoral findings were primarily widespread erosions and ulcerations. Three patients had skin lesions during the course of the disease. The patients’ histories of persistent lesions for several months in addition to the fact that the patients denied the occurrence of prodromal symptoms or a new drug intake calls into question the tentative EM diagnosis. Thus, PV was considered another differential diagnosis. Few studies have reported similar findings of lip lesions among PV patients [22–25].

This case series included three young adults and three older adults and included five females and one male all of middle eastern decent. The most common age of PV presentation has been reported to be between the 4th and 6th decades [26]. A female predominance was reported in a study conducted in Kuwait [27], whereas an equal gender distribution was noticed among PV patients from Finland [28]. On the other hand, a few other studies have reported male predominance [29, 30]. The female predominance suggests a hormonal relationship [31]. In the Arab world, PV is the most common subtype in Egypt, Sudan, Morocco, Syria, Kuwait, Saudi Arabia and Yemen [32]. PV is well known to be prevalent among specific ethnic groups, such as individuals of Ashkenazi Jewish and Mediterranean ancestries [33]. Interestingly, Kridin and coworkers have shown that the Arab population was affected by PV at a younger age compared to the Jewish population, which could be explained by the genetic susceptibility associated with HLA types [33].

In the current series, incisional biopsies from the perilesional mucosa were taken from all six patients demonstrating acantholysis (Tzank cells), intraepithelial clefting and a tombstone pattern, which was suggestive of PV. DIF was performed for Cases 1, 2 and 3, demonstrating intercellular deposition of IgG (Cases 1 and 2) and both IgG and C3 (Case 3).

The aim of treating patients with PV is to achieve rapid control of the disease, followed by consolidation and maintenance phases [1]. According to the British Association of Dermatologists, the management of PV has two phases. The induction phase controls disease activity, and the maintenance phase prevents relapse with minimal drug concentration [33]. Systemic corticosteroid dose calculation is based on disease severity. For mild PV cases, a steroid dose of 0.5–1 mg/kg/day is commonly used, whereas treatment of severe PV cases may be initiated at 1–2 mg/kg/day [34]. Once PV lesions are controlled, the systemic steroid dose may be tapered [34]. Systemic steroids are frequently combined with other immunosuppressants, such as azathioprine, mycophenolate mofetil, cyclophosphamide, methotrexate, immunomodulators such as high-dose intravenous immunoglobulin (IVIG), or antibody-eliminating procedures such as immunoadsorption [33]. Oral lesions can also be managed with topical steroids in the form of an oral gel or mouthwash [35]. Intralesional steroid therapy may be indicated for refractory oral lesions [35]. All patients in this case series were managed with systemic steroids, i.e., prednisolone 0.5-1 mg/kg/day, together with steroid-sparing agents and topical steroid mouthwashes.

This case series was limited by the fact that DIF was not performed in all the patients. Such investigation requires specific facilities that are not readily available in all health care services. Furthermore, in the case of test availability in the private sector, the cost may not be affordable for most of the patients who were originally seen in governmental or educational hospitals. Although DIF was not performed in three cases, the routine histopathological examination of all specimens in this series was consistent with classic PV histology, as described earlier in the literature.

In conclusion, this case series emphasizes that oral PV may be misdiagnosed as EM in a subgroup of patients who present with persistent lip hemorrhage and crusting. Misdiagnosing PV as EM affects the treatment plan and outcome of the patients. EM is a self-limiting condition that requires supportive care in most cases and perhaps a short course of treatment. In contrast, PV requires long-term treatment to control disease activity and minimize relapse. Therefore, a comprehensive history, clinical examination and incisional biopsies should be considered in such patients.

## Data Availability

The datasets used and/or analysed during the current study are available from the corresponding author upon reasonable request.
